# A review of electrochemical oxidation technology for advanced treatment of medical wastewater

**DOI:** 10.3389/fchem.2022.1002038

**Published:** 2022-09-15

**Authors:** Chengyu Zhang, Zhisheng Yu, Xiangyang Wang

**Affiliations:** ^1^ College of Resources and Environment, University of Chinese Academy of Sciences, Beijing, China; ^2^ Binzhou Institute of Technology, Weiqiao-UCAS Science and Technology Park, Binzhou, Shandong Province, China; ^3^ RCEES-IMCAS-UCAS Joint-Lab of Microbial Technology for Environmental Science, Beijing, China

**Keywords:** electrochemical oxidation, electrode materials, electrochemical reactor, medical wastewater, advanced treatment

## Abstract

Antibiotics widely exist in medical wastewater, which seriously endanger human health. With the spread of the COVID-19 and monkeypox around the world, a large number of antibiotics have been abused and discharged. How to realize the green and efficient treatment of medical wastewater has become a hot research topic. As a common electrochemical water treatment technology, electrochemical oxidation technology (EOT) could effectively achieve advanced treatment of medical wastewater. Since entering the 21st century, electrochemical oxidation water treatment technology has received more and more attention due to its green, efficient, and easy-to-operate advantages. In this study, the research progress of EOT for the treatment of medical wastewater was reviewed, including the exploration of reaction mechanism, the preparation of functional electrode materials, combining multiple technologies, and the design of high-efficiency reactors. The conclusion and outlook of EOT for medical wastewater treatment were proposed. It is expected that the review could provide prospects and guidance for EOT to treat medical wastewater.

## Introduction

**FIGURE 1:**
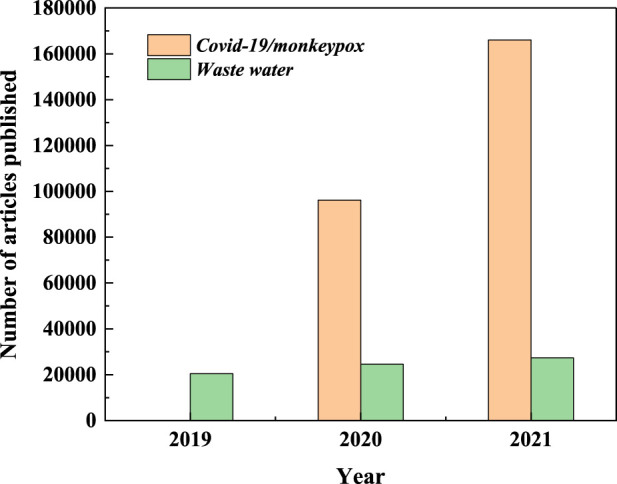
Papers related to Covid-19/Monkeypox virus and waste water.

Antibiotics are one of the ubiquitous refractory organic pollutants (ROPs) in the water environment, and have attracted widespread attention due to their long-term residual, semi-volatile, and high toxicity (Scott et al., 2016). In particular, a large number of antibiotics exist in medical wastewater, and the concentration could reach the level of mg/L (Khan et al., 2020). The high toxicity and resistance to degradation of antibiotics could seriously endanger human health after entering the environment (Patangia et al., 2022). Trace amounts of antibiotics in the environment could increase the resistance of microorganisms, which leads to the development of drug-resistant superbugs (Sandegren, 2019). In addition, the number of papers related to the advanced treatment of medical wastewater continues to increase with the spread of the COVID-19 and monkeypox virus around the world (Figure 1). Therefore, it is urgent to find a green and efficient method to remove antibiotics in aqueous solution.

Currently, EOT is gradually becoming a favorable technology for degrading antibiotics from wastewater due to the mineralization of antibiotics into carbon dioxide and water (Lima et al., 2020). In particular, EOT has the advantages of green, high efficiency, easy operation, and mild reaction conditions (Xue et al., 2021). In addition, EOT could carry out biochemical and disinfection treatment of medical wastewater at the same time, which effectively cuts off the transmission path of pathogens (Lei et al., 2020). It has been widely concerned by researchers because of its unique application value. At present, the reaction rate of EOT was mainly improved through the development of functional electrode materials (Shestakova and Sillanpää, 2017). In addition, electrochemical oxidation water treatment technology could achieve deep purification of low-concentration medical wastewater by combining with photocatalytic technology and membrane treatment technology (Cho et al., 2019).

In this paper, the current research progress of EOT in medical wastewater treatment was reviewed. The advantages of EOT in water treatment are highlighted, and new research directions for EOT to treat medical wastewater were proposed, including functional electrode materials, multi-technology integration, and electrochemical reactors. Overall, this review provided new ideas for the development and engineering application of electrochemical oxidation water treatment technology.

## Electrochemical indirect oxidation

Electrochemical indirect oxidation exhibited unique advantages compared with electrochemical direct oxidation. For example, the rate of migration of contaminants from the solution to the electrode surface would not be inhibited; the oxidation reaction on the anode surface would increase the electrochemical oxidation rate. In particular, a large amount of reactive intermediates (•OH, •O^2−^) was generated in the anode during the reaction process, and the medical wastewater was treated advanced through a single electron transfer process (Garcia-Segura et al., 2018).

### Reactive intermediates

Hydroxyl radicals (•OH), active chlorine, chlorine-containing oxidants, etc. are the main reactive intermediates in the electrochemical indirect oxidation process. In particular, •OH exhibits stronger oxidative properties (E = 2.8V) compared to others (Miao et al., 2020). The generation of chemisorbed •OH was attributed to lattice oxygen in the anode material (Xu et al., 2022). However, physisorbed •OH was generated through the reaction between electrons and water molecules (or OH^−^) (Martínez-Huitle and *Brillas*, 2009). In addition, active chlorine species, including •Cl, Cl_2_, and HClO, were efficiently generated when electrochemical oxidation was used to treat chlorine-containing wastewater (Huang et al., 2020). Also, high health risk by-products chlorite and perchlorate were generated (Hao et al., 2022).

$$Cl\to \;•Cl+e^{-}$$ (1)

$$2Cl\to Cl_{2}+2e^{-}$$ (2)

$$Cl_{2}\to \;•OH\to HClO+Cl^{-}$$ (3)

### Anode material

The electrode material not only affected the oxygen evolution overpotential of the electrode, but also determined the amount and type of active intermediates generated. C*hemisorbed* •*OH* was generated on the surface of active electrodes (such as ruthenium, titanium) and interacted with the electrodes to generate superoxide. Moreover, the refractory organic pollutants were selectively oxidized into small molecular substances (Nakayama and Honda, 2021). However, *physisorbed* •*OH* was generated on the surface of inactive electrodes (such as Pb, Sn, Sb)*,* which could mineralize organic matter into carbon dioxide and water (Mameda et al., 2020). At present, Dória et al. (2021) and Zhu et al. (2021) presented active electrodes such as Ti/RuO^2−^Sb_2_O_4_ and Ti/RuO_2_-ItO_2_ have been developed, indicating that the stability of active anodes could be effectively improved by loading metal oxides. The stability and activity of inactive anode materials were enhanced by means of doping or intercalation (Rai and Sinha, 2022). The oxidation rate of chlorinated hydrocarbons was increased by 60% when using the inactive anode compared to the active anode, which might be attributed to the free radicals generated by the inactive anode (Zhang et al., 2016). Interestingly, the removal efficiency of organics by the active electrode was significantly higher than that of the inactive electrode in the treatment of chlorinated wastewater. This might be attributed to the higher instantaneous current efficiency of metal active electrodes compared with graphite electrodes and diamond electrodes (Kraft et al., 2002). As reported, the generation efficiency of active chlorine of Ru, Ir, and Pt electrodes is significantly higher than that of inactive electrodes (such as PbO_2_ and SnO_2_) (Sirés et al., 2014). Zinc oxide-coated electrodes could effectively degrade perfluorinated compounds in wastewater, removing 66% of pollutants within 40 min (Zhang et al., 2016). Therefore, active electrodes were preferentially used in the treatment of chlorine-containing wastewater.

## Mediated electrochemical oxidation

**FIGURE 2:**
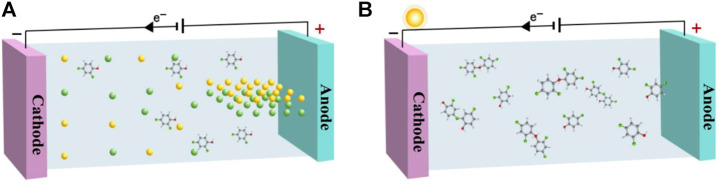
**(A)** Mediated electrochemical oxidation system, **(B)** Photo/electrochemical oxidation system.

Mediated electrochemical oxidation degrades organic pollutants by reversible redox couples. The redox substances could not only be recycled, but also could avoid the surface of the anode material from being polluted by organic substances (Figure 2A). Ag^2+^ is a strong oxidant and could react with halide ions to form precipitates during water treatment, thereby reducing the rate of electrochemical oxidation (Kakiuchi and Samec, 2020). As reported, Co^3+^ as an oxidant could not only improve the kinetics of the oxidation reaction, but also effectively avoid the formation of precipitates (Sequeira et al., 2006). Ce^4+^ acted as a medium to participate in the electrochemical oxidation process, and the solution pH, electrolyte, and current density affected the oxidation rate of cerium and then changed the degradation rate of phenolic pollutants (Matheswaran et al., 2008). In particular, Ce^4+^ exhibited excellent stability during the reaction. Moreover, the reaction temperature, reaction time, and reactor flow rate were all factors that affected the rate of the mediated electrochemical oxidation reaction (Balaji et al., 2008). Overall, the mediated oxidation process could be enhanced by adding redox substances, thereby improving the removal efficiency of pollutants in the treatment of actual wastewater.

## Photo/electrochemical oxidation

Photo/electrochemical oxidation is a photoresponse that generates holes and electrons under illumination conditions, which are enhanced by the action of an electric field (Figure 2B). TiO_2_ is currently a commonly used photocatalyst, and electron-holes are generated by electron transitions from the valence band (VB) to the conduction band (CB) under UV (<380 nm) light irradiation (Liu et al., 2019a). Reactive oxygen species (ROS) could be efficiently generated by single-electron transfer of photogenerated electrons (holes), thereby decomposing organic pollutants (Tao et al., 2020). In addition, the applied electric field could effectively inhibit the recombination of electron-holes and accelerate the generation of ROS. The photo-assisted electrochemical system was constructed using TiO_2_ as the electrode material, which improved the degradation efficiency of bisphenol A (Liu et al., 2019b). The TiO_2_/Ti electrode exhibited good stability under low current density conditions and was suitable for application in a three-electrode system. It has been reported that the TiO_2_/Ti electrode could effectively degrade pollutants under UV irradiation, which was attributed to the generation of ROS (Carneiro et al., 2004). Due to the stability at high current density, Ru_0.3_Ti_0.7_O_2_/Ti electrodes were widely used in two-electrode systems. The degradation efficiency of total organic carbon (TOC) increased by 50% in the Ru_0.3_Ti_0.7_O_2_/Ti/UV system, which might be due to the photoelectric synergistic generation of ·OH (Hussain et al., 2017). In particular, BiOCl/NaNbO_3_ nanomaterials exhibited high current density and electron-hole separation efficiency due to their special bandgap position and layered structure (Li et al., 2022). Overall, the photo/electrochemical oxidation synergistic system could effectively improve the degradation efficiency of wastewater.

## Electrochemical reactor

**FIGURE 3:**
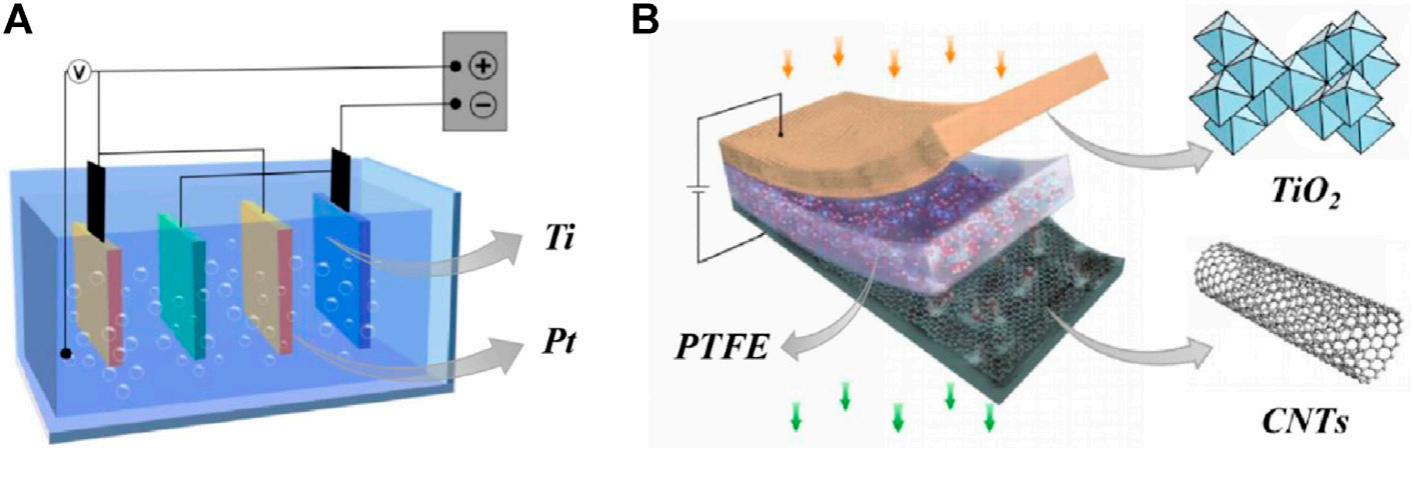
**(A)** Submerged electrochemical reactor system, **(B)**Penetrating electrochemical reactor system.

The electron transfer rate and the degradation efficiency of organic pollutants during the reaction are not only related to the electrode material, but also to the configuration of the reactor. At present, the commonly used electrochemical reactors are submerged and penetrating. Due to the free diffusion method, the submerged electrochemical reactor had the disadvantages of low current efficiency and low mass transfer rate, and could not treat low-concentration wastewater (Figure 3A). In particular, penetrating electrochemical reactors exhibited numerous advantages due to their unique sandwich structure, including small plate spacing, high electron transfer rate, and low energy consumption (Figure 3B) (Wangwang et al., 2019). In comparison to the submerged electrochemical reactor, Santos et al. (2014) showed the degradation efficiency of pollutants increased by 200% using the penetrating electrochemical reactor, which was attributed to its sandwich reactor structure. Using carbon-based catalysts as electrocatalytic anodes, the degradation efficiency of pollutants was increased by 600% in the penetrating electrochemical reactor, which was due to the efficient mass transfer efficiency (Han et al., 2012). A novel type of flow reactor was invented, and the degradation efficiency of synergistic electrochemical oxidation of levofloxacin increased by 95% (Pieczyńska et al., 2019). A tubular electrode reactor was designed to enhance electrochemical wastewater treatment, which was attributed to the titanium oxide (M-TiSO) anode and superior reactor configuration (Liang et al., 2021). The construction of an electric Fenton/double anode reactor system significantly improved the degradation efficiency of bisphenol A, which was attributed to the slow release of Fe^2+^ (Zhao et al., 2022). Also, this indicated a good synergistic effect between the electric Fenton and the double anode reactor. A penetrating electrochemical reactor was constructed using graphene as the electrode material to treat chlorine-containing wastewater and exhibited good removal efficiency, which was attributed to the HClO generated during the reaction (Liu et al., 2014). Moreover, the rate of electrochemical oxidation reaction was affected by the mode of power supply. Compared with DC power supply, pulse power supply not only improved the utilization rate of electric energy, but also effectively reduced the deposition of organic matter on the anode surface. As reported, the degradation efficiency was increased by 80% using the pulse power supply method, and the effects of the initial pollutant concentration and current density were investigated (Jiang et al., 2021). Overall, the penetrating electrochemical reactor in the pulse power supply mode and the electric fenton could effectively improve the treatment efficiency of wastewater.

## Conclusion and outlook

With the development of material technology and electrochemical technology, the research work on electrochemical oxidation water treatment technology showed a continuous and in-depth trend. Novel electrode materials and electrochemical reactors were constantly being developed, forming a green, efficient, and energy-saving electrochemical oxidation water treatment technology. In addition, the research on the joint application of various technologies had made great progress. A large number of articles discussed the application of EOT in water treatment, but most of them remained in the laboratory stage. Various problems still exist in the application process and need to be solved urgently. The main research directions in the future were proposed.

1) Preparation of high-performance electrode materials. High-performance electrode materials could effectively improve the rate of interfacial electron transfer and mass transfer, which was the key to electrochemical water oxidation treatment technology. Furthermore, the economics of electrode materials should be considered during practical engineering applications.
2) Optimization of electrochemical reactors. Excellent electrochemical reactors could effectively improve the efficiency of water treatment and the utilization of electricity by interface regulation. Also, the design of the electrochemical reactor should consider the ease of operation in practical applications.
3) Combined application of multiple technologies. By the combination of electric Fenton, light, media and EOT, the synergy between technologies could be exerted to improve the efficiency of water treatment. Moreover, potential environmental risks should be considered in the combined application of multiple technologies.

## Data Availability

The original contributions presented in the study are included in the article/Supplementary Material, further inquiries can be directed to the corresponding author.
